# Anabolic–Androgenic Steroids Induced Cardiomyopathy: A Narrative Review of the Literature

**DOI:** 10.3390/biomedicines13092190

**Published:** 2025-09-07

**Authors:** Panagiotis Iliakis, Eleftheria Stamou, Alexandros Kasiakogias, Eleni Manta, Athanasios Sakalidis, Angeliki Vakka, Panagiotis Theofilis, Freideriki Eleni Kourti, Dimitrios Konstantinidis, Kyriakos Dimitriadis, Charalambos Vlachopoulos, Costas Tsioufis

**Affiliations:** First Department of Cardiology, School of Medicine, National and Kapodistrian University of Athens, Hippokration General Hospital, 115 27 Athens, Greece; eleftheriastamou1@gmail.com (E.S.); akasiakogias@gmail.com (A.K.); asakalidis@gmail.com (A.S.); aggvakka@outlook.com (A.V.); panos.theofilis@hotmail.com (P.T.); frylenakourtii@gmail.com (F.E.K.); kon_dimitris@hotmail.com (D.K.); dimitriadiskyr@yahoo.gr (K.D.); cvlachop@otenet.gr (C.V.); ktsioufis@gmail.com (C.T.)

**Keywords:** anabolic steroids, cardiomyopathy, sports cardiology

## Abstract

Anabolic–androgenic steroids (AASs) are synthetic derivatives of testosterone and are increasingly misused to enhance muscle growth and physical performance, particularly among athletes and recreational bodybuilders. Although AASs affect multiple organ systems, their severe and potentially life-threatening complications involve the cardiovascular system. This review summarizes current knowledge on the pathophysiological mechanisms and clinical manifestations of AAS-induced cardiomyopathy. Chronic supraphysiologic AAS use promotes cardiac injury and adverse cardiac remodeling via oxidative stress, androgen receptor overactivation, RAAS dysregulation, and pro-apoptotic signaling. These changes could lead to hypertension, dyslipidemia and atherosclerosis, myocardial fibrosis and hypertrophy, arrhythmias, heart failure, and kidney injury. Vascular dysfunction, increased arterial stiffness, and a prothrombotic state further compound the cardiovascular risks. Diagnostic approaches involve biomarker evaluation, echocardiography, and cardiac magnetic resonance imaging, revealing structural and functional cardiac abnormalities such as reduced ejection fraction, concentric hypertrophy, myocardial fibrosis, and impaired diastolic function. Although cessation of AAS use may lead to partial or complete reversal of cardiac dysfunction in some individuals, others may experience irreversible myocardial damage. The reversibility appears to depend on dosage, duration of exposure, and early intervention. This review explores the cardiovascular consequences of AAS use, with a focus on the mechanisms, diagnosis, and management of AAS-induced cardiomyopathy, and underlines the importance of education and early detection.

## 1. Introduction

Anabolic–androgenic steroids (AASs) are synthetic compounds derived from testosterone, the primary male sex hormone. In humans, testosterone is naturally produced by the Leydig cells in the testes. It is commonly administered in esterified forms as a therapeutic treatment for male hypogonadism. Additionally, testosterone and certain other AASs, such as nandrolone and oxandrolone, may be prescribed for medical conditions like osteoporosis or aplastic anemia. Since the 1950s, AASs have been misused by athletes in competitive sports due to their performance-enhancing and strength-boosting effects. In the 1980s, this misuse had extended beyond professional athletes to amateur and recreational users in gym settings. Although AASs have legitimate medical applications, they are frequently abused in doses significantly higher than those used in clinical practice to increase muscle growth strength [1,2]. The use of AASs may enhance strength when combined with resistance exercises such as bench presses and weightlifting; however, these drugs do not significantly impact aerobic performance [3]. In a randomized, controlled trial, healthy men were administered 600 mg of testosterone enanthate or a placebo weekly for 10 weeks. The results showed that men who received testosterone without exercise experienced significant increases in muscle size and strength compared to the placebo group. Those who combined testosterone with resistance training had even greater improvements, gaining an average of 6.1 kg in fat-free mass and demonstrating notable increases in quadriceps and triceps’ muscle size. Muscle strength improved significantly in the testosterone groups, with the combination of testosterone and exercise producing the greatest gains [4]. Additionally, a systematic review and meta-analysis found that AAS use in athletes leads to a moderate increase in lean body mass and a 52% greater improvement in strength compared to placebo [5].

The use of anabolic–androgenic steroids (AASs) is associated with multiple adverse effects across various body systems [1]. Cardiovascular complications include hypertension, dyslipidemia, and an increased risk of heart disease [2]. Endocrine disruptions can lead to testosterone suppression, infertility, and gynecomastia [6]. Hepatic effects, particularly with oral AAS, may result in liver toxicity. Psychological effects, such as aggression, mood swings, and dependence, are also reported [2]. Additionally, AAS use can cause skin issues like acne, increased body hair, and male-pattern baldness. Long-term misuse may further contribute to kidney damage and increased risk of thrombosis [1,2].

In recent years, AAS abuse has been increasingly linked to severe cardiovascular events in healthy young athletes. Reported complications include cardiomyopathy, atrial fibrillation, stroke, myocardial infarction, disturbances in the hemostatic system, thrombosis, and systemic embolism [7,8]. Case reports have also associated AAS use with acute heart failure, sudden cardiac death, ventricular fibrillation during exercise, cardiac tamponade, and dilated cardiomyopathy [1,9]. There is no robust evidence regarding AAS-induced cardiomyopathy’s prevalence; however, a small cross-sectional study of 101 weightlifting AAS users demonstrated that approximately 11% of the population had reduced ejection fraction, while a nationwide cohort study tracking AAS users (*n* = 1189) over ~11 years against age- and sex-matched controls (*n* = 59,450) demonstrated that AAS users were nearly nine times more likely to develop cardiomyopathy than non-users (adjusted hazard ratio (aHR) of 8.90 (95% CI: 4.99–15.88)) [10,11].

Resistance training has gained popularity among both men and women seeking to maintain or improve their physical fitness [12]. However, the use of AASs for performance enhancement poses significant health risks. Increased awareness among both the general population and healthcare professionals is crucial for effective diagnosis, treatment, and prevention. This narrative review aims to provide an updated overview of AAS abuse, its mechanisms of action, its role in AAS-induced cardiomyopathy, and its current medical management approaches; the relevant literature was identified primarily through searches in PubMed and Scopus up to May 2025, using combinations of keywords such as “anabolic–androgenic steroids,” “cardiomyopathy,” “cardiac remodeling,” “hypertrophy,” “heart failure,” and “arrhythmia.” Both preclinical (animal and in vitro) studies and clinical evidence (case reports, case series, cohort studies, and reviews) were included if they addressed the cardiovascular effects of anabolic steroid use. Given the narrative nature of this review, no formal inclusion/exclusion criteria or quantitative synthesis were applied.

## 2. Pathophysiology

Anabolic–androgenic steroids exert their effects primarily through androgen receptor (AR) activation, influencing multiple physiological systems [13]. When administered at supraphysiological doses, AASs disrupt endogenous testosterone biosynthesis and interact with various signaling pathways beyond AR activation. High levels of AASs antagonize glucocorticoid receptors, reducing protein catabolism and enhancing muscle growth through the growth hormone (GH) and insulin-like growth factor-1 (IGF-1) axis [14]. Their impact on oxidative stress is significant, as AASs disrupt mitochondrial respiratory chain function, increasing reactive oxygen species (ROS) production, which contributes to DNA damage and tissue injury. This oxidative imbalance has been linked to impairments in endothelial function, neurotoxicity, and cardiac ischemic injury. Furthermore, AASs induce apoptosis, triggering cellular death in the brain, kidneys, and vascular smooth muscle cells [15,16]. In the cardiovascular system, renin–angiotensin–aldosterone system (RAAS) overactivation plays a crucial role in AAS-induced cardiac remodeling. Excessive aldosterone secretion promotes left ventricular (LV) hypertrophy, fibrosis, and arrhythmias, increasing the risk of serious cardiac conditions independent of blood pressure levels. The presence of AAS metabolites in urine has even been suggested as a potential predictor of cardiac abnormalities in AAS users [17].

### 2.1. Atherosclerosis 

The use of AASs in supraphysiological doses has been linked to alterations in plasma lipoprotein levels [6,18,19]. Atherosclerosis is directly associated with dyslipidemia, characterized by increased low-density lipoprotein (LDL) levels, decreased high-density lipoprotein (HDL) levels, altered lipoprotein (a) concentrations, and reduced apolipoprotein A (ApoA) levels [20]. The impact of AASs on serum lipid profiles is dose-dependent, and these lipid alterations typically hinder the regression of atherosclerotic plaques [21,22]. Prolonged high-dose AAS abuse can lead to atherosclerosis and, consequently, coronary heart disease, cerebrovascular disease, or peripheral arterial occlusive disease [23]. Moreover, long-term AAS abuse has been linked to increased homocysteine blood levels, contributing to hyperhomocysteinemia, a known risk factor for coronary atherosclerosis and, ultimately, coronary artery disease (CAD) [24,25]. A cross-sectional study on AAS use in humans and mice found increased PWV, reduced carotid compliance, and higher cIMT in AAS users, indicating arterial stiffening and early atherosclerosis. Similarly, testosterone-treated mice showed aortic compliance loss, suggesting AAS-induced vascular dysfunction and heightened cardiovascular risk [26]. Other studies and case reports link AAS use to stroke, peripheral vascular disease, and hypertension [27,28,29,30].

### 2.2. Hypertension

The relationship between AAS use and blood pressure (BP) remains inconclusive. While some studies have reported a correlation between AAS use and elevated BP [19,31,32], others have found no significant association [33,34]. One study observed that current AAS users had higher BP compared to former users and non-using weightlifters [31]. Another study reported a significant BP increase after an 8-week AAS cycle (*p* < 0.01), reaching hypertensive levels, though BP returned to normal 8 weeks post-cycle [35]. Additionally, current AAS abusers exhibit elevated 24 h systolic BP, including overt hypertension, while both current and former users are characterized by increased aortic stiffness compared to controls [32]. Evidence also suggests that anabolic steroid use raises both systolic and diastolic BP, with the extent of the increase correlating with the duration of abuse [28]. Furthermore, research by Junior et al. demonstrated that AAS users did not experience a significant post-exercise reduction in arterial pressure, suggesting that AASs may impair post-exercise hypotension [36]. The precise mechanism remains unclear, but it has been proposed that AASs may elevate BP through excessive adrenal production of 11-β-deoxycorticosterone or by stimulating renal renin secretion [28,37]. A study by Rosca et al. in rats showed that nandrolone decanoate (DECA) significantly increased systolic blood pressure (SBP) and plasma angiotensin-converting enzyme (ACE) activity while reducing nitric oxide (NOx) metabolites, thus supporting evidence that androgens activate the renin–angiotensin system, contributing to hypertension [18].

### 2.3. Myocardial Apoptosis

Growing evidence suggests that AASs are associated with myocardial apoptosis [38]. An experimental study on adult rat ventricular myocytes demonstrated that AAS exposure induces dose-dependent apoptosis, with stanozolol, testosterone enanthate, and testosterone increasing apoptotic cell death and upregulating the pro-apoptotic oncogene Bax [39]. Hassan et al. conducted a controlled study in rats, revealing a significant increase in cardiac caspase-3 activity in nandrolone decanoate-treated rats, along with histological evidence of apoptosis and myocyte hypertrophy [40]. Additionally, research highlights the interplay between testosterone, angiotensin II, and tumor necrosis factor-alpha (TNF-α) in apoptosis induction. Increased caspase-3 activity in cultured cardiomyocytes suggests that this process reduces cell viability, demonstrating the cytotoxic effects of high testosterone concentrations in a dose-dependent manner [41]. Similarly, histological analysis of Wistar rat hearts exposed to high-dose testosterone enanthate showed myocardial hypertrophy, fibrosis, and collagen deposition. Electron microscopy revealed mitochondrial edema, disorganized sarcomeres, and endothelial damage, while caspase-3 staining confirmed apoptosis. These findings underscore the cardiotoxic effects of AAS abuse, further reinforcing its role in cardiac remodeling, fibrosis, and cell death [42].

### 2.4. Vasospasm

Androgen receptors (ARs) are widely expressed in the coronary arterial wall, making this vascular bed particularly susceptible to AAS effects. While physiological testosterone levels promote vasodilation by inhibiting voltage-gated calcium channels and stimulating calcium-dependent potassium channels, supraphysiological doses upregulate calcium currents, leading to vasoconstriction [43]. Additionally, AASs increase circulating vasoconstrictive factors, such as norepinephrine, angiotensin II, and thromboxane while downregulating vasodilatory factors [43,44,45]. Chronic AAS use also induces vascular remodeling, characterized by smooth muscle cell hypertrophy, further predisposing to vasospasm [43]. Clinical evidence supports these findings, with reports of AAS users experiencing severe chest pain and myocardial ischemia despite normal coronary angiograms [46]. AAS-induced vasospasm may be particularly dangerous in individuals with coronary artery disease (CAD), where reduced androgen receptor expression could exacerbate vascular constriction. This sudden vasoconstriction can trigger atherosclerotic plaque rupture and thrombosis, potentially leading to myocardial infarction [47].

### 2.5. Thrombosis

AAS use significantly increases the risk of thrombosis by affecting platelet function, the coagulation/fibrinolysis system, and vascular tone [48]. These substances enhance platelet aggregation at sites of endothelial injury and stimulate the production of procoagulant factors, contributing to a hypercoagulable state [23,49]. They also increase thromboxane A2 (TXA2) levels, which suppress prostacyclin, a key inhibitor of platelet aggregation [19,50]. Additionally, AASs modify arterial structure by reducing elastin while increasing collagen and fibrous proteins, leading to arterial stiffness [26,51]. These changes heighten the likelihood of arterial embolism, deep vein thrombosis, and pulmonary embolism [52]. The combined effects of AAS-induced vascular dysfunction and hypercoagulability can accelerate cardiovascular complications, predisposing users to early-onset myocardial infarction (STEMI or NSTEMI) [53,54]. However, further studies are needed to determine whether this prothrombotic risk persists after AAS cessation.

### 2.6. Cardiac Hypertrophy

Anabolic–androgenic steroid (AAS) abuse promotes excessive cardiac tissue growth, leading to secondary hypertrophy resembling hypertrophic cardiomyopathy, apoptotic cell death, and ventricular remodeling. These changes increase the risk of myocardial infarction, cardiomyopathy, and sudden cardiac death (SCD), even in the absence of coronary thrombosis or atherosclerosis [55,56]. The hypertrophic effects of AASs stem from direct androgen receptor stimulation, with severity depending on dose and duration of use [57]. Additionally, AASs disrupt the RAAS axis, which contributes to LV hypertrophy and fibrosis through elevated blood pressure, angiotensin II action on cardiac myocytes via the angiotensin type 1 (AT-1) receptor, and aldosterone-mediated effects [58]. Long-term AAS exposure is associated with both systolic and diastolic dysfunction; increased LV mass; thicker ventricular walls; and reduced LV ejection fraction, sometimes below normal limits [53]. AAS users also exhibit lower right ventricular global longitudinal strain and elevated systolic blood pressure [11,59]. Animal studies demonstrate severe cardiac injury, including myocardial degeneration, striation loss, sarcomere disruption, and mitochondrial damage [60]. Testosterone induces cardiomyocyte hypertrophy via mammalian target of rapamycin complex 1/ribosomal protein S6 kinase 1 (mTORC1/S6K1) axis [61]. AAS use also heightens susceptibility to myocardial ischemia/reperfusion injury, impairing postischemic function and increasing infarct size [62]. High-dose nandrolone decanoate in young rabbits induces fibrosis, inflammation, oxidative stress, and reduced catalase activity. It also alters the Myocardial Performance Index (MPI), indicating diastolic dysfunction despite preserved systolic function. Notably, subcutaneous nandrolone administration has a prolonged harmful impact, with oxidative stress and biochemical markers failing to normalize even after discontinuation [63]. It is important that the aforementioned studies be interpreted with caution due to relevance limitations to human physiology.

### 2.7. Kidney Injury

Anabolic–androgenic steroids negatively impact kidney function, contributing to acute kidney injury, chronic kidney disease (CKD), and glomerular toxicity. These effects are linked to RAAS activation, increased endothelin levels, oxidative stress, and elevated pro-fibrotic (TGF-β1) and inflammatory cytokines (IL-1β, IL-6, and TNF-α) [64]. The presence of androgen receptors (ARs) in kidney tissue facilitates cell growth and hypertrophy, particularly in the proximal and distal convoluted tubules. Long-term nandrolone exposure may promote fibrosis through AR activation [16,65]. Furthermore, AAS misuse combined with a high-protein diet has been associated with severe renal conditions, such nephroangiosclerosis, focal segmental glomerulosclerosis (FSGS), and interstitial nephritis [66]. Table 1 shows some of the commonly used AASs, their route of administration, and some key features.

A substantial portion of the mechanistic understanding of AAS-induced cardiomyopathy is derived from preclinical animal models, which provide valuable insights into pathways such as myocardial fibrosis, oxidative stress, apoptosis, and altered calcium handling. However, important limitations must be acknowledged. Animal studies often involve supraphysiologic dosing protocols and short exposure periods that may not accurately reflect human AAS-use patterns, which are typically prolonged, cyclical, and combined with other substances. Moreover, species-specific differences in cardiac physiology and steroid metabolism complicate direct extrapolation. For these reasons, translational emphasis is essential. Clinical evidence—particularly cardiac MRI studies demonstrating myocardial fibrosis, and echocardiographic findings of impaired systolic and diastolic function in long-term users—supports the relevance of these experimental mechanisms to human disease. Thus, while animal data elucidate biological plausibility, their greatest value lies in complementing human observational studies and helping to explain the structural and functional cardiac abnormalities reported in AAS users.

## 3. Diagnosis of AAS-Induced Cardiomyopathies

Diagnosing AAS-associated cardiomyopathy requires a comprehensive step-by-step approach. This includes obtaining a detailed history of AAS use, performing laboratory tests to measure AAS levels when feasible, and employing imaging techniques and invasive procedures to evaluate cardiac function. A study showed that individuals who tested positive for AAS use had a threefold increased risk of developing non-ischemic heart disease, including cardiomyopathy and atrial fibrillation, compared to matched control subjects (HR, 2.9; 95% CI, 1.7–5.0) [67]. Patients with anabolic steroid-induced cardiomyopathy typically present with symptoms such as shortness of breath, palpitations, lower-limb edema, chest pain, orthopnea, and occasionally syncope [68,69,70]. These symptoms, often related to heart failure or arrhythmias, are what usually prompt them to seek medical care. In some cases, the first sign may be a serious event like atrial fibrillation or stroke [7,27,29].

### 3.1. Biomarkers

In patients with anabolic steroid-induced cardiomyopathy, several biomarkers can be assessed to evaluate cardiac function and detect myocardial injury. Standard cardiac biomarkers such as troponins (cTnT and cTnI), B-type natriuretic peptide (BNP), and N-terminal pro-BNP (NT-proBNP) are commonly used to assess myocardial injury and the severity of heart failure. Elevated troponin levels indicate myocardial necrosis and have been associated with increased LV mass, late gadolinium enhancement (LGE) on cardiac MRI, global myocardial strain, and maximal LV thickness on echocardiography. Similarly, increased BNP or NT-proBNP levels reflect myocardial wall stretch and correlate with LV fibrosis on cardiac MRI [71,72]. However, specific data on these elevations in the context of anabolic steroid-induced cardiomyopathy remain limited. Long-term use of AASs is also linked to elevated inflammatory markers, such as interleukin-8 (IL-8) and extracellular matrix (ECM)-remodeling enzymes like matrix metalloproteinase-9 (MMP-9), both of which have been associated with myocardial dysfunction, particularly in current users, indicating an ongoing risk of cardiac damage [73]. Additionally, AAS use is known to cause dyslipidemia, characterized by increased low-density lipoprotein (LDL) and decreased high-density lipoprotein (HDL), further contributing to cardiovascular risk [20,74].

### 3.2. TTE

Cardiac dysfunction associated with anabolic–androgenic steroid (AAS) use—including ischemia, LV hypertrophy, and reduced ejection fraction (EF)—can be assessed using several modalities. Transthoracic echocardiography (TTE) is commonly employed to assess left ventricular ejection fraction (LVEF), LV systolic strain, biventricular size, and conventional indices of diastolic function [59]. Doppler echocardiography and tissue Doppler imaging (TDI) have demonstrated impaired LV relaxation in AAS users. For example, Nottin et al. reported prolonged isovolumetric relaxation time and reduced Em/Am ratios at the mitral annulus, while Krieg et al. found lower Em/Am ratios at the basal interventricular septum in steroid users compared to controls, indicating diastolic dysfunction [75,76]. Baggish et al. studied long-term weightlifting AAS users and found that 71% of current users had LVEF values below the normal threshold of 52%, and 50% had early diastolic mitral annular velocity (E′) values below 8.5 cm/s. These users also exhibited increased LV mass index, thicker LV walls, and more concentric geometry, whereas former users showed partial recovery [53]. Additionally, a cross-sectional study comparing 101 AAS users with 71 non-using weightlifting controls found that AAS users had significantly higher LV mass index (106 ± 26 vs. 80 ± 15 g/m^2^), lower LVEF (49 ± 7% vs. 59 ± 5%), impaired right ventricular global longitudinal strain (− 17.3 ± 3.5 vs. − 22.8 ± 2.0%), and higher systolic blood pressure [11]. The HAARLEM study observed a 5% decline in LVEF after an AAS cycle, along with increased LV mass, wall thickness, and left atrial (LA) volume. A decline in the E/A ratio by 0.45 further indicated reduced LV compliance and diastolic dysfunction [77]. D’Andrea et al., using speckle tracking echocardiography (STE) and cardiopulmonary exercise testing, demonstrated increased LA volume index and reduced LA systolic strain in AAS users, suggesting LA enlargement and impaired myocardial deformation. LA strain also correlated with reduced exercise capacity, highlighting its impact on cardiovascular performance [78]. Another cross-sectional study confirmed that AAS users had greater LA enlargement, higher LA stiffness, and impaired reservoir and conduit functions compared to controls [79].

### 3.3. ECG

Beyond structural changes, long-term AAS use alters myocardial electrophysiology. Users often present with post-exercise ECG abnormalities, such as prolonged QRS duration, atrial fibrillation, and ventricular arrhythmias. Compared to non-users, AAS-using bodybuilders showed increased inter- and intra-atrial electromechanical delay and prolonged repolarization, as reflected by an elevated Tp-e interval, Tp-e/QT ratio, and Tp-e/QTc ratio [23,80].

### 3.4. Cardiac MRI

Cardiac magnetic resonance imaging (CMR) offers a comprehensive, non-invasive assessment of cardiac structure, function, perfusion abnormalities, inflammation, and fibrosis in individuals using anabolic–androgenic steroids (AASs) [81]. In contrast to echocardiography, which can be limited by acoustic window quality and relies on geometric assumptions, CMR delivers highly accurate and reproducible measurements of ventricular volumes, mass, and function. Moreover, it allows for tissue characterization and detection of focal fibrosis through late gadolinium enhancement imaging. These advantages establish CMR as the gold standard for detecting subtle myocardial alterations associated with AAS use [82]. CMR in a 39-year-old bodybuilder with a 20-year history of AAS use revealed myocardial scarring alongside severe LV hypertrophy, despite the presence of normal coronary arteries [81]. Another report described a 31-year-old bodybuilder presenting with acute heart failure secondary to dilated cardiomyopathy and severe, irreversible LV systolic dysfunction (LVEF 23%), where CMR revealed a small area of late gadolinium enhancement (LGE), significant LV dilation, and severe biventricular dysfunction (LVEF 18%, RV ejection fraction 22%) [83]. In contrast, a different case showed low–normal biventricular function and mildly increased myocardial mass after medical therapy, without evidence of inflammation or scarring [84].

Angell et al. used CMR to study male bodybuilders actively using AASs and found significant increases in LV mass and wall thickness compared to non-users. Additionally, they observed a reduction in right ventricular ejection fraction and impaired LV longitudinal strain, indicating early signs of myocardial dysfunction. However, despite these pathological structural and functional changes, no evidence of focal myocardial fibrosis was detected by LGE imaging. These findings suggest that although CMR is highly sensitive in detecting ventricular remodeling and systolic dysfunction, its ability to identify focal fibrosis in AAS users is limited, potentially due to the diffuse or microscopic nature of the fibrotic changes [82]. Rasmussen et al. similarly used CMR to assess current and former AAS users and found that current users exhibited concentric LV hypertrophy, reduced left and right ventricular ejection fractions, and an increased LV mass/EDV ratio compared to controls. Again, no detectable fibrosis was found with LGE or T1 mapping techniques, reinforcing the hypothesis that detectable fibrosis may not be a frequent finding among asymptomatic AAS users [85].

Luijkx et al., using CMR, showed that AAS users demonstrated significantly lower biventricular ejection fractions and decreased E/A ratios compared to athletes and non-athletes who did not use steroids. Linear regression analyses confirmed that AAS use was independently associated with adverse changes in ventricular volumes, mass, and diastolic parameters [86]. Finally, Ismail et al. reported that anabolic steroid use among bodybuilders can lead to significant LV hypertrophy, with patterns of remodeling that may resemble hypertrophic cardiomyopathy. Consequently, anabolic steroid use should be considered in the differential diagnosis of unexplained LV hypertrophy, particularly in athletes [82]. In a subsequent study, Ismail et al. utilized cardiac magnetic resonance imaging (CMR) to assess myocardial perfusion in steroid-using bodybuilders. Their findings confirmed the presence of marked LV hypertrophy and revealed alterations in resting myocardial blood flow distribution compared to non-users. Despite these abnormalities at rest, hyperemic perfusion during stress remained comparable between groups, and no focal myocardial fibrosis was detected. These observations suggest that anabolic steroid use may impair resting microvascular function without significantly affecting stress-induced perfusion [87].

## 4. Treatment of AAS-Induced Cardiomyopathies

Management of anabolic-induced cardiomyopathy demands tailored therapeutic strategies alongside careful cardiovascular risk mitigation to prevent adverse events, including treatment of induced cardiomyopathy, as well as preventing sudden cardiac death (SCD) [10,88]. The therapeutical cornerstone is the immediate and complete cessation of anabolic steroid usage. Discontinuation of AAS overuse can halt further myocardial injury and, in some cases, reverse cardiac dysfunction [88,89]. Many reports indicate partial or complete recovery of LV function after stopping AASs and starting heart failure therapy [10,21,88]. Moreover, evidence-based, guideline-directed management of heart failure is of great importance and indicated for all patients with AAS-induced cardiomyopathy, in the same manner as with other causes of heart failure [90,91]. In the case of reduced systolic function, the main pillars of treatment include b-blockers, which could reduce sympathetic stimulation, prevent arrhythmias, and improve mortality [21,89]; angiotensin-converting enzyme inhibitors (ACEIs) or angiotensin receptor blockers (ARBs), which could reverse ventricular remodeling and reduce mortality and morbidity; angiotensin receptor–neprilysin inhibitors (ARNis) with further mortality reduction; and mineralocorticoid receptor antagonists (MRAs), such as spironolactone or eplerenone, which confer additional survival benefits and SGLT2 inhibitors with documented benefit in hospitalizations and all-cause mortality in the whole spectrum of systolic function [21]. Diuretics could be utilized, especially upon the presence of overload symptoms, congestion, and increased LV filling pressures, but with no documented mortality benefit [21]. In the rare case that AAS cessation, as well as guideline-directed HF treatment, does not lead to functional improvement, if indicated, advanced heart failure therapies, such as mechanical circulatory support, or even cardiac transplantation (on the basis of the young age of this population) could be considered by the treating physicians.

Alongside guideline-directed treatment, management of cardiovascular risk factors is of high importance, including aggressive control of hypertension, diabetes, and dyslipidemia [10]. Non-pharmacological therapies, including smoking cessation, structured physical activity programs tailored to each patient’s risk and needs, and advocating for a healthy diet, should be at the center of a modern, personalized therapeutical approach [10].

Heart failure, especially when accompanied by the presence of reduced ejection fraction, is associated with increased risk of sudden cardiac death (SCD). There are no specific guideline-directed recommendations for the prevention of SCD in this population; however, given that AAS use is associated with myocardial fibrosis, electrical instability, and increased arrhythmic risk, regular Holter monitoring and advanced cardiac imaging with MRI may be warranted in these individuals for risk stratification [10,92]. Nevertheless, when patients meet standard criteria for primary or secondary SCD prevention as per established heart failure guidelines, they should be considered candidates for ICD implantation [10,21]. These criteria include an ejection fraction persistently ≤35% despite at least 3 months of optimal medical therapy, or survivors of prior cardiac arrest or documented sustained ventricular arrhythmias [88]. If there are documented atrial or ventricular arrhythmias, beta-blockers are generally first-line medical treatment for rate or rhythm control, and catheter ablation could be considered for drug-refractory arrhythmias [21]. It is of great importance to note that the avoidance of proarrhythmic drugs is prudent, particularly given the underlying substrate in AAS-induced disease.

Most importantly, lifestyle changes and educational interventions targeting the hazards of AAS use—including the risk of SCD—should be included in personalized treatment discussions, at which there should be a bidirectional doctor–patient relationship, moving towards improving desired outcomes. Referral to addiction or behavioral health services is recommended in cases of established or suspected AAS dependence [88,89].

## 5. Reversibility of AAS-Induced Cardiomyopathies

The reversibility of anabolic–androgenic steroid (AAS)-induced cardiomyopathy remains a topic of clinical debate. While some individuals experience significant improvement in cardiac function after discontinuing AAS use, others sustain persistent myocardial damage that may necessitate lifelong management [68]. Several studies and case reports illustrate a wide spectrum of outcomes, suggesting that the degree of reversibility may depend on factors such as cumulative dosage, duration of use, and time to intervention. Urhausen et al. have used echocardiography to show that, even after 12 months of abstinence, former AAS-using bodybuilders exhibited residual concentric LV hypertrophy [31]. Similarly, a cross-sectional cohort study found that long-term AAS users had both systolic and diastolic dysfunction, along with increased coronary artery plaque burden compared to nonusers. Notably, systolic function tended to normalize after drug cessation, whereas diastolic dysfunction and atherosclerotic changes appeared less reversible [53]. Rasmussen et al. also reported reduced systolic function in both current and former AAS users, reinforcing the notion that cardiovascular toxicity may not fully resolve after drug discontinuation [85]. Another study showed severe biventricular cardiomyopathy and pathologically elevated systolic blood pressure persisting in former AAS users, and only partial regression of ventricular impairment was documented. A history of AAS use emerged as the strongest independent predictor of adverse cardiac remodeling [11]. Conversely, the HAARLEM study provided evidence that some structural and functional cardiac changes can be resolved fully after cessation, particularly in individuals with shorter or less intensive AAS exposure [77]. A supporting case report described complete cardiac recovery following guideline-directed therapy in a patient with AAS-induced cardiomyopathy [84]. However, other case reports describe irreversible cardiomyopathy years after AAS use, reinforcing the notion that chronic exposure can lead to permanent cardiac damage [83,93]. These findings underscore the complexity of AAS-related cardiac pathology and highlight the need for individualized long-term cardiovascular monitoring.

## 6. Limitations of the Current Evidence Base

While the current body of literature consistently suggests a link between anabolic–androgenic steroid (AAS) use and cardiomyopathy, several limitations must be acknowledged when interpreting these findings. First, most available data arise from small observational cohorts, case series, or individual case reports. Such designs are prone to selection bias and incomplete data capture, and they lack the statistical power to adjust for potential confounders, such as concurrent substance use, comorbidities, or training intensity. Second, there is marked heterogeneity across studies with respect to the populations examined (elite athletes vs. recreational users vs. patients with pre-existing cardiovascular risk factors), the type and duration of AAS exposure, and the diagnostic methods employed to define cardiomyopathy (echocardiography, cardiac MRI, or histopathology). These differences complicate direct comparisons and may partly explain the variability in reported prevalence and severity of cardiac dysfunction. Third, while preclinical animal studies provide valuable mechanistic insights into pathways such as oxidative stress, fibrosis, and altered calcium handling, their translational relevance is limited by differences in dosing regimens, metabolism, and the controlled experimental setting. Collectively, these methodological constraints underscore the need for cautious interpretation: the available evidence supports an association but falls short of proving causality. Large, prospective, longitudinal studies with standardized diagnostic criteria are required to more definitively characterize the risk, natural history, and reversibility of AAS-induced cardiomyopathy.

## 7. Conclusions

Anabolic–androgenic steroid overuse represents a major health issue of increasing importance, with profound implications for cardiovascular health, particularly the development of cardiomyopathy in young individuals without prior cardiac disease. Diagnostic evaluation should combine clinical history with biomarkers, echocardiography, and cardiac MRI for comprehensive assessment, given the spectrum of structural and functional abnormalities seen in AAS users. Management centers on immediate cessation of AAS use, which may lead to partial or complete reversal of cardiac dysfunction, particularly when identified early and accompanied by guideline-directed medical therapy for heart failure, including beta-blockers, RAAS inhibitors, mineralocorticoid receptor antagonists, and SGLT2 inhibitors. The potential for irreversible myocardial injury in chronic or high-dose users underscores the necessity for early detection and intervention, alongside lifestyle modifications and multidisciplinary care to address underlying substance misuse. Future research should aim to elucidate the precise mechanisms and thresholds of AAS-induced cardiac injury, the long-term outcomes post-cessation, and effective educational interventions to prevent AAS misuse. Increased awareness among clinicians and the public is critical to mitigate the cardiovascular harms associated with AAS abuse. By fostering an individualized approach, healthcare systems can better address the burden of AAS-induced cardiomyopathy, aiming to reduce morbidity, mortality, and healthcare costs associated with this preventable condition.

## Figures and Tables

**Table 1:** Commonly used AASs [2,67].

Compound	Route of Administration	Key Features
**Methandrostenolone/** **methandienone**	Oral	Increase muscle strength; fluid retention; prostatic hypertrophy; withdrawn
**Stanozolol**	Oral/injectable	Treatment of hereditary angioedema; preserve lean mass; hepatotoxicity; veterinary medicine
**Oxymetholone**	Oral	Treatment of anemia; extremely potent; significant weight gain; hepatotoxicity; carcinogenicity; withdrawn
**Methyltestosterone**	Oral	Androgen replacement therapy; antineoplastic agent; mood swings; hepatotoxicity; withdrawn
**Danazol**	Oral	Anti-estrogen; treatment of endometriosis, breast fibrocystic disease; weight gain; edema
**Fluoxymesterone**	Oral	Treatment of delayed puberty in males, breast neoplasms in women; very potent; hepatotoxicity; cardiovascular complications
**Norethandrolone**	Oral	Clinical trial phase III acute myeloid leukemia; treatment of aplastic anemia, Fanconi anemia
**Oxandrolone**	Oral	Used in hypogonadal men, in HIV-wasting syndrome, in burns; weight gain–muscle strength; liver toxicity
**Methylepitiostanol (Epistane)**	Oral	Weight loss–athletic performance; hepatotoxicity; infertility; withdrawn
**Testosterone (cypionate, enanthate, and propionate)**	Injectable	Treatment of male hypogonadism; longer half-life; hypertension
**Nandrolone decanoate**	Injectable	Treatment of anemia of renal insufficiency, senile and postmenopausal osteoporosis; virilization; dyslipidemia; reduced libido
**Boldenone undecanoate**	Injectable	Illegal for human use; veterinary steroid; weight gain
**Methenolone acetate/enanthate**	Oral/injectable	Treatment of anemia caused by bone marrow failure and muscle-wasting conditions; fluid and electrolyte retention; virilization

## Data Availability

As this is a review article, no data were created.

## Abbreviations

AASs	Anabolic androgenic steroids
ACEIs	Angiotensin-converting enzyme inhibitors
AR	Androgen receptor
ARBs	Angiotensin receptor blockers
BP	Blood pressure
CAD	Coronary artery disease
CKD	Chronic kidney disease
CMR	Cardiac magnetic resonance imaging
DECA	Nandrolone decanoate
EF	Ejection fraction
GH	Growth hormone
LA	Left atrium
LGE	Late gadolinium enhancement
LV	Left ventricular
LVEF	Left ventricular ejection fraction
MPI	Myocardial Performance Index
PWV	Pulse wave velocity
RAAS	Renin–angiotensin–aldosterone system
SBP	Systolic blood pressure
SCD	Sudden cardiac death
STE	Speckle tracking echocardiography
TDI	Tissue Doppler imaging
TTE	Transthoracic echocardiography
